# A phase I trial of pembrolizumab with hypofractionated radiotherapy in patients with metastatic solid tumours

**DOI:** 10.1038/s41416-018-0281-9

**Published:** 2018-10-15

**Authors:** Amit Maity, Rosemarie Mick, Alexander C. Huang, Sangeeth M. George, Michael D. Farwell, John N. Lukens, Abigail T. Berman, Tara C. Mitchell, Josh Bauml, Lynn M. Schuchter, Mark O’Hara, Lilie L. Lin, Angela Demichele, John P. Christodouleas, Naomi B. Haas, Dana M. Patsch, Stephen M. Hahn, Andy J. Minn, E. John Wherry, Robert H. Vonderheide

**Affiliations:** 1 Department of Radiation Oncology, Perelman School of Medicine, University of Pennsylvania, Philadelphia, PA USA; 2 Abramson Cancer Center, Perelman School of Medicine, University of Pennsylvania, Philadelphia, PA USA; 3 Department of Biostatistics, Epidemiology and Informatics, Perelman School of Medicine, University of Pennsylvania, Philadelphia, PA USA; 4 Department of Medicine, Perelman School of Medicine, University of Pennsylvania, Philadelphia, PA USA; 5 Institute for Immunology, Perelman School of Medicine, University of Pennsylvania, Philadelphia, PA USA; 6 Department of Radiology, Perelman School of Medicine, University of Pennsylvania, Philadelphia, PA USA; 7 Division of Radiation Oncology, University of Texas MD Anderson Cancer Center, Houston, TX USA; 8 Department of Microbiology, Perelman School of Medicine, University of Pennsylvania, Philadelphia, PA USA

**Keywords:** Cancer immunotherapy, Radiotherapy

## Abstract

**Background:**

We conducted a phase I trial evaluating pembrolizumab+hypofractionated radiotherapy (HFRT) for patients with metastatic cancers.

**Methods:**

There were two strata (12 patients each): (i) NSCLC/melanoma progressing on prior anti-PD-1 therapy, (ii) other cancer types; anti-PD-1-naive. Patients received 6 cycles of pembrolizumab, starting 1 week before HFRT. Patients had ≥2 lesions; only one was irradiated (8 Gy × 3 for first half; 17 Gy × 1 for second half in each stratum) and the other(s) followed for response.

**Results:**

Of the 24 patients, 20 (83%) had treatment-related adverse events (AEs) (all grade 1 or 2). There were eight grade 3 AEs, none treatment related. There were no dose-limiting toxicities or grade 4/5 AEs. Stratum 1: two patients (of 12) with progression on prior PD-1 blockade experienced prolonged responses (9.2 and 28.1 months). Stratum 2: one patient experienced a complete response and two had prolonged stable disease (7.4 and 7.0 months). Immune profiling demonstrated that anti-PD-1 therapy and radiation induced a consistent increase in the proliferation marker Ki67 in PD-1-expressing CD8 T cells.

**Conclusions:**

HFRT was well tolerated with pembrolizumab, and in some patients with metastatic NSCLC or melanoma, it reinvigorated a systemic response despite previous progression on anti-PD-1 therapy. Clinical Trial Registration: NCT02303990 (www.clinicaltrials.gov).

## Background

Immune checkpoint blockade (ICB) has demonstrated success in treating patients with certain types of metastatic cancer^1^. One of the immune checkpoints that has been most successfully targeted is the PD-1 receptor, found on the surface of T cells^2^. Under healthy conditions, PD-1 acts to down-regulate excessive immune responses upon engagement of its ligands, programmed death-ligand 1 or 2 (PD-L1 or PD-L2). The PD-1 receptor–ligand interaction is also a major pathway hijacked by tumours enabling them to evade immune surveillance. Antibodies that block the PD-L1/PD-1 interaction diminish the downregulation of the anti-tumour immune response, potentiating the cytotoxic function of tumour-specific T cells. Multiple drugs that inhibit the PD-1/PD-L1 axis have been approved by the Food and Drug Administration (FDA) for treatment of various cancers in the metastatic setting^1^. In spite of these successes, objective response rates remain disappointing and many responders will eventually progress through treatment.

Emerging evidence indicates that radiotherapy (RT) can stimulate the immune response. Diverse mechanisms have been proposed for this immune stimulation^3–5^, including increased major histocompatibility complex class I expression on tumour cells^6^, increased type I interferon production by tumour cells^7^ and increased activation of dendritic cells and presentation of tumour-specific antigens to draining lymph nodes^8^.^9^^,^ Recent work from our institution suggests that cell cycle progression through mitosis following double-stranded DNA breaks leads to the formation of micronuclei which precede activation of inflammatory signalling^10^. In animal models RT has also been shown to potentiate systemic response to immunomodulatory Flt-3 ligand therapy, a major growth factor stimulating dendritic cells^11^, and immune checkpoint blockade^4^. There are also reports of patients on ICB who have received radiation and then experienced disease regression in regions *outside* the radiation fields^12,13^, the so-called “abscopal effect”. Our own group has published both pre-clinical data and results of a phase I trial combining RT with the anti-CTLA4 antibody ipilimumab, suggesting a benefit to the addition of RT^14^. Our group and others have shown in mouse models that adding RT to anti-PD-1 therapy can increase the efficacy of the immunotherapy^14–19^.

Based on these data, we designed a phase I trial to investigate the combination of pembrolizumab with hypofractionated radiotherapy (HFRT) using 1–3 large doses of radiation. Recognising the difficulty of distinguishing the synergistic role of HFRT versus the effect of PD-1 blockade alone, we specifically included patients with progression on PD-1 blockade. We have completed the Safety Phase of this trial and continue to enrol patients onto the Expansion Phase. In this paper we report the toxicity and initial efficacy outcomes of the Safety Phase.

## Materials and methods

### Study design

This was an open-label, phase I study. All patients were recruited and treated at a single centre, the Perelman Center for Advanced Medicine (PCAM), which houses the Abramson Cancer Center of the University of Pennsylvania. Our objectives were to define dose-limiting toxicities (DLTs) and identify tolerable schedules of radiation in combination with pembrolizumab. A secondary objective was to assess the treatment response to the combination of pembrolizumab with HFRT in non-index (i.e., non-irradiated) metastatic lesions. Lastly, an exploratory objective was to evaluate the immune pharmacodynamic changes in the peripheral blood after this combination therapy.

**Fig. 1:**
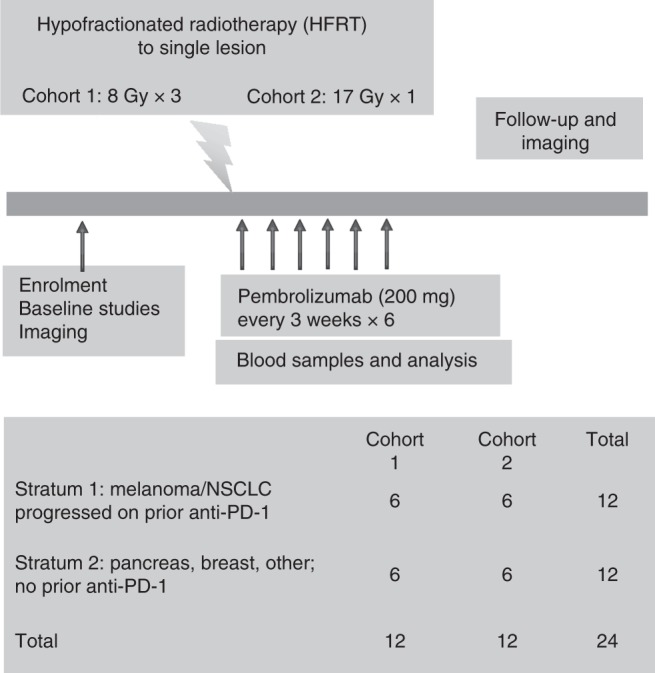
Trial schema and stratification. Patients were stratified by histology and whether they had received prior PD-1 or PDl-1 therapy. Within each stratum, the first six patients received 8 Gy × 3 to a single lesion and the second six patients received 17 Gy × 1

Figure 1 shows the trial schema. Pembrolizumab was administered at a fixed dose of 200 mg intravenously every 3 weeks beginning 1 week prior to the first fractionation of radiation. Although many patients were treated using a stereotactic body radiotherapy (SBRT) technique, the protocol did not require this technique, and some patients were treated using a three-dimensional conformal technique or electrons. Patients were enrolled into one of two strata based on histology and prior therapy. Stratum 1 consisted of patients with metastatic melanoma or non-small cell lung cancer (NSCLC) who had progressed on a prior PD-1 or PD-L1 therapy. At the time of trial design, these were the only cancers with an FDA approval for anti-PD-1/PD-L1 therapy. Stratum 2 consisted of patients who had other cancers and had not received prior anti-PD-1 or PD-L1 therapy. A total of 12 patients were enrolled per stratum. In each stratum the first 6 patients received 8 Gy × 3 fractions to a single target lesion, and the next 6 patients received 17 Gy × 1 fraction. The intent of this design was to explore two different fractionation schedules, and not to perform an escalation of radiation dose. Subsequent doses of pembrolizumab were administered every 3 weeks for a total of 6 doses. The protocol was subsequently amended so that patients who had completed 6 doses of pembrolizumab and were doing well could continue on the drug.

### Patients and eligibility criteria

The protocol stipulated that pancreatic cancer patients could have locally advanced or metastatic disease but for all other disease sites, only stage IV patients were eligible. In fact, all pancreatic cancer patients who ended up being enrolled had metastatic disease.

Patients were required to have an index lesion ≥1 cm that was amenable to HFRT and at least one other lesion that was not irradiated and could be followed for response using RECIST 1.1 (Response Evaluation Criteria in Solid Tumors 1.1). This lesion, if it was close to the radiated lesion, could have received no more than 10% of the dose prescribed to the target lesion. Stratum 1 was eligible for patients with melanoma or NSCLC who had received at least 2 doses of an anti-PD-1 or anti-PD-L1 therapy and had progressive disease documented radiologically by RECIST v1.1 criteria. For stratum 2, those patients with breast or pancreatic cancer must have had progression or refractory disease to at least one regimen of therapy for metastatic disease.

A few of the key exclusion criteria were the presence of active central nervous system metastases, autoimmune disease and immunodeficiency.

### Toxicity assessment

DLT was defined as any grade 3 or higher non-haematological toxicity and any grade 4 haematological toxicity that is probably, possibly or definitely related to the combination of pembrolizumab and HFRT. All acute toxicities were graded using the National Cancer Institute (NCI) Common Terminology Criteria for Adverse Events v4.0 (CTCAE). Immune-related toxicity was defined as an adverse event that is associated with exposure to pembrolizumab and that is consistent with an immune adverse event. Radiotherapy–related toxicity was defined as any toxicity observed within the tissues contained within the radiation portal and was consistent with tissue reaction to radiation exposure at any time during follow-up. Follow-up for toxicity was performed to day 196 from first pembrolizumab administration.

### Response assessment

A baseline radiological tumour assessment was conducted prior to the start of any therapy using computed tomography (CT), F-fluorodeoxyglucose positron emission tomography/computed tomography (FDG PET/CT), or magnetic resonance imaging. The first on-study radiological assessment of tumour response status was performed at day 64 (cycle 4 of pembrolizumab), unless there was clinical indication warranting earlier radiologic imaging. If imaging at 64 days showed progressive disease (PD), treatment with pembrolizumab could be continued until a repeat assessment 4–6 weeks later confirmed PD. If the repeat scan confirmed PD, the date of disease progression was recorded based on the prior scan in calculating progression-free survival (PFS).

In patients who continued on trial, subsequent radiologic imaging was performed 30 days and 90 days after cycle 6 of pembrolizumab.

RECIST 1.1 was used to assess overall response by aggregating the largest axial diameter of non-irradiated target lesions^20^. A maximum of non-irradiated 5 target lesions in total were identified at baseline and measured throughout the course of therapy. The percent change in volume of the irradiated (index) lesion was also calculated, but this was not used in the RECIST measurement.

### Immunohistochemical staining

PD-L1 staining was performed by an outside company (QualTek Molecular Laboratories, Newtown, PA, USA) using the Dako 22-C3 antibody. PD-L1 staining was scored as 0, 1+, 2+ or 3+. H-score was calculated as ((1 × % cells with 1+ staining) + (2 × % cells with 2+ staining) + (3 × % cells with 3+ staining)). A modified Proportion Score (MPS) was also calculated as overall percent of cells expressing PD-L1.

### Human peripheral blood and lymphocyte isolation

Peripheral blood mononuclear cells (PBMCs) were obtained only from patients treated at the Abramson Cancer Center of the University of Pennsylvania after signed, informed consent for an additional protocol allowing phlebotomy of patients enrolled on the treatment study. The protocol was approved by the institutional review board at the University of Pennsylvania. Absolute lymphocyte count was obtained from a complete blood count and differential as measured by an accredited clinical lab. PBMCs were obtained by Ficoll centrifugation (Amersham Pharmacia Biotech; Piscataway, NJ, USA) and viably frozen at −50 °C until use.

### Pharmacodynamic assessments: flow cytometry

Peripheral blood samples to measure CD8 T lymphocyte activation were obtained at baseline and at defined times during the course of the study. Cryopreserved PBMC samples from pre-treatment and post-treatment time points were thawed and stained with master mix of antibodies for surface stains including CD8 (ebioscience, San Diego, CA; RPA-T8), PD-1 (Biolegend, San Diego, CA, USA; EH12.2H7), CD45RA (Biolegend; HI100), CD27(BD, San Diego, CA, USA; L128) and intracellular stains for CTLA4 (BD; BNI3), Eomes (ebioscience; WD1928), Tbet (biolegend; 4B10) and Ki67(BD; B56). Permeabilisation was performed using the Foxp3 Fixation/Permeabilisation Concentrate and Diluent kit (eBioscience). Cells were resuspended in 1% paraformaldehyde until acquisition on a BD Biosciences LSR II cytometer and analysed using FlowJo (Tree Star, San Diego, CA, USA).

### Statistical methods

In the safety phase, the DLT observation window was defined from the first infusion of pembrolizumab to 35 days after the final radiation fraction. Toxicity was scored by NCI CTCAE v4.0. A fractionation cohort was considered tolerable if one or fewer DLTs were observed in 6 patients within the cohort. If 2 or more DLTs were observed, then enrolment was stopped for that cohort. If 2 or more DLTs were observed in cohort 1, then enrolment proceeded to cohort 2, since cohort 2 is a different radiation schedule and not considered an escalation of radiation dose. Each stratum was evaluated separately for safety.

Patients were scored for response using RECIST criteria as: complete response (CR), partial response (PR), stable disease (SD) or progressive disease (PD). Two patients exhibited rapid progressive disease after 1 month on treatment and for whom progression could not be documented from clinical exam or imaging. They were considered not evaluable (i.e., PD/NE). One additional patient was lost to follow-up at approximately 6 weeks without any disease evaluation and was considered not evaluable (i.e., lost/NE). PFS was defined from first infusion of pembrolizumab to first documented progression of disease or death due to any cause. Patients who were alive and without progression were censored on the last date that documented their progression-free status (i.e., clinic or imaging date). Overall survival (OS) was defined from first infusion of pembrolizumab to death due to any cause or last patient contact alive.

Plans for data analysis included toxicity grading and tabulation by stratum and fractionation cohort. Baseline patient characteristics were summarised by stratum, including mean, standard deviation and range for continuous variables and frequency and percentage for categorical variables. Event rates and exact 95% confidence intervals were calculated. A comprehensive tabulation of tumour and treatment characteristics and clinical outcomes for patients was displayed by stratum and fractionation cohort. PFS and OS were computed in months for each patient.

## Results

### Patient characteristics

From March 2015 until April 2016, 24 patients with metastatic solid tumours, 12 in each stratum, were enrolled and treated (Table 1). The average age was 60 years, 41.7% of patients were male and all enrolling patients were Caucasian. Supplementary Figure 1 shows the Consort diagram which details the number of patients who were referred in the safety phase and how many were enrolled. Out of 45 patients who were referred, 8 ended up pursuing alternative therapy/trial, 2 died of rapid progression of disease and 11 were found to be ineligible for our trial. The reasons for ineligibility included: patients with pancreatic cancer who had not yet received and progressed on first-line chemotherapy (*n* = 4), lack of disease outside of index lesion that could be followed for abscopal response (*n* = 2), newly discovered brain metastasis (*n* = 1), treatment with high-dose steroids (*n* = 1) and unsuitability of lesion to undergo HFRT due to location or prior treatment with radiation (*n* = 3).

**Table 1:** Patient characteristics by stratum

Characteristic	Stratum 1, progressed on PD-1/PD-L1, *n* = 12		Stratum 2, no prior PD-1/PD-L1, *n* = 12		Total, *n* = 24	
Age, mean + SD (range)	61.7 ± 14.3 (34–84)		57.7 ± 8.4 (40–68)		59.7 ± 11.7 (34–84)	
	No.	%	No.	%	No.	%
Site of primary disease						
NSCLC	8	67	0	0	8	33
Melanoma	4	33	0	0	4	17
Pancreas	0	0	4	33	4	17
Breast	0	0	4	33	4	17
Head and neck	0	0	1	8	1	4
Renal cell Ca	0	0	2	17	2	8
Colon	0	0	1	8	1	4
Gender			3			
Male	7	58	9	25	10	42
Female	5	42	75	75	14	58
Ethnicity						
Caucasian	12	100	12	100	24	100
ECOG performance status						
0	6	50	8	67	14	58
1	6	50	4	33	10	42
Site of target (irradiated lesion)						
Lung	3	25	3	25	6	25
Liver	1	8	8	67	9	38
Soft tissue	6	50	0	0	6	25
Renal or adrenal	2	17	0	0	2	8
Bone	0	0	1	8	1	4
Previous therapy						
Chemotherapy	8	67	10	83	18	75
Anti-CTLA-4	3	25	1	8	4	17
Anti-PD-1	12	100	0	0	12	50
Surgery	9	75	9	75	18	75
Radiation	8	67	7	58	15	63

By trial design, stratum 1 consisted of patients with metastatic NSCLC (*n* = 8) or melanoma (*n* = 4) who had progressed on prior PD-1 or PD-L1 therapy (Supplementary Table 1). This table (last column) details the reasons why these patients were classified as having progressed. Ten of the 12 patients in stratum 1 were deemed to have progressed because of the detection of new lesions radiologically. In the other 2 patients, progression was documented by an increase in the size of lesions radiologically (44 and 39%).

Supplementary Table 1 also shows the number of cycles of anti-PD-1 therapy patients had received prior to being enrolled on our RadVax trial. The number of cycles ranged from 4 to 22 with a median of 9.5. Eight patients had received pembrolizumab, three had received nivolumab and one had received ipilimumab/nivolumab and subsequently pembrolizumab.

Stratum 2 consisted of patients with a variety of tumour histologies, including: pancreas (*n* = 4), breast (*n* = 4), renal cell carcinoma (*n* = 2), head and neck (*n* = 1) and colon (*n* = 1). None of these patients had received prior PD-1 therapy, and 10 of the 12 patients had received previous chemotherapy (Supplementary Table 1).

The location of the index lesion that was irradiated was at the discretion of the treating radiation oncologist and included lung, liver, soft tissue site, adrenal and bone. In stratum 1, soft tissue (50%) and lung (25%) were most frequently irradiated sites, while in stratum 2, the majority of patients received radiation to liver lesions (67%).

### Toxicity

All adverse events (AEs) recorded on the trial are summarised in Supplementary Table 2, regardless of their attribution. The table also shows which AEs were felt to possibly or probably relate to pembrolizumab (P) or radiation (R). Importantly, there were no DLTs related to the combination of radiation and pembrolizumab. There were no grade 4 or 5 toxicities.

Out of 24 patients, 20 (83.3%) had treatment-related AEs (attributed to pembrolizumab and/or radiation). In 17 of these 20 patients (70.8%), the AEs were specifically attributed to pembrolizumab (all grades 1–2). The most common treatment-related AEs were nausea, vomiting, diarrhoea, constipation, fever, fatigue and weakness. Eleven of the 20 patients with treatment-related AEs were in stratum 1 and the remaining in stratum 2.

There were eight grade 3 AEs, none attributable to either radiation or pembrolizumab. These included two cases of weakness and one each of bacteraemia, small bowel obstruction, fever, dehydration, confusion and hypotension. There were two immune-related adverse events (hypothyroidism and pneumonitis), both grade 2, which occurred in the same patient (#16) who received radiation to a lung metastasis. The pneumonitis that this patient developed was treated with steroids, and his clinical course was similar to that seen in most patients with radiation pneumonitis. He remains alive 31.4 months after radiation with no evidence of disease, no pulmonary symptoms (cough, dyspnoea, etc.) and no limitation in physical activity. Two other patients had a history of immune events while they were receiving anti-PD-1 therapy prior to enrolling on this study (hence not included in table). Patient 27 had developed immune-mediated nephritis while on pembrolizumab previously which had resolved at the time of study enrolment, and patient 32 had developed grade 2 colitis while on nivolumab which had lessened to grade 1 by the time he was enrolled on our study. Of note, neither of these patients developed these toxicities again while on the pembro-HFRT trial.

In addition to the patient discussed above with pneumonitis, which was attributed to both radiation and pembrolizumab, three other patients had grade 1 radiation-related AEs. Two patients developed transient abdominal pain after receiving abdominal radiation, one of whom also developed a self-limited episode of nausea and vomiting. A third patient developed transient scalp pain after being irradiated to that area.

### Radiologic response

In 11 of the 24 patients there was a decrease in the size of the irradiated (index) lesion. In five patients there was an increase. There were eight cases in which the change in the volume of the index tumour were not evaluable; in three there were no restaging images available, in two the restaging images did not include the irradiated lesion, in two the radiation changes obscured the index tumour, and in one case the index lesion was an osseous metastasis, hence response was not measurable.

**Fig. 2:**
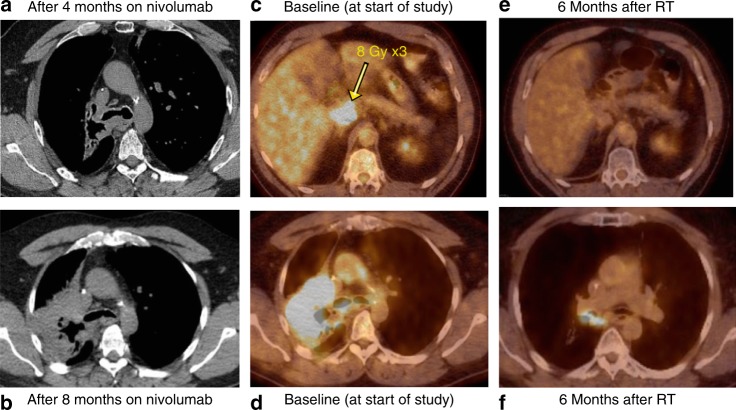
Images for responder with non-small cell lung cancer. Patient 15 was diagnosed with non-small cell lung cancer metastatic to the bone and was given carboplatin/paclitaxel x 6 cycles with a good response and then given palliative radiation to the right lung mass (37.5 Gy). He developed a mass in the ileum and received gemcitabine/navelbine but progressed. He received nivolumab to which he had a good response initially. (**a**) Chest CT scan after 4 months on nivolumab. CT scan 2 months later showed progression of disease in the chest. CT scan done 2 months after this showed further progression of disease (**b**). At this time, nivolumab was discontinued, and he was enrolled on our pembrolizumab HFRT study. He underwent a planning FDG PET/CT scan that established his baseline disease status (**c**, **d**). He received pembrolizumab and then radiation (8 Gy × 3) to an abdominal mass (**c**) followed by continued pembrolizumab. A repeat PET/CT scan was performed 6 months after radiation (**e**, **f**) showing response in non-irradiated lesions which had previously progressed on PD-1 blockade prior to study entry

In terms of our secondary objective, which was to assess the treatment response to the non-index (non-irradiated) metastatic lesions, in stratum 1, two patients (#15 and 27) had a PR for an overall response rate of 16.7% (Table 2). Notably, both patients experienced regression at sites outside of the irradiation fields after prior progression on anti-PD-1 therapy (Fig. 2 and Supplementary Figure 2). Both experienced prolonged PFS after being treated on the pembro-HFRT protocol (9.2 and 28.1 months). In stratum 2, one patient with renal cell carcinoma experienced a CR (#16), and 2 patients (#3 and 9) experienced prolonged SD (7.4 months for a patient with adenoid cystic carcinoma and 7.0 months for another patient with renal cell carcinoma). The overall response rate for the entire study population was thus 12.5% (3 out of 24). It is noteworthy that out of the two patients in stratum 1 who had radiologic response after radiation and pembrolizumab (#15 and 27) and the one patient in stratum 2 who had a CR (#16), two of them had an immune-related toxicity either on the trial (#16) or with prior anti-PD-1 therapy (#27).

**Table 2:** Tumour and treatment characteristics and clinical outcomes by stratum and fractionation

No.	Primary site	PD-L1/H-score/ MPS^a^	Target lesion site	Target lesion size (cc)	Pembro no. of cycles^b^	Pembro delay or dose modification	% Change in index lesion volume^c^	RECIST response^d^	PFS (months)	OS (months)
Stratum 1, 8 Gy × 3										
2	NSCLC	ND	Adrenal	140.8	2	No	26.2	PD	1.6	1.7
14	Melanoma	ND	Liver	5.5	3	No	Imaging not available	Lost/NE	1.4^f^	1.4^f^
15	NSCLC	ND	Nodal	16.2	6 (+8)^e^	No	–50.00%	PR	9.2	19.7
17	NSCLC	ND	Renal	20.5	5	No	−27.8	PD	1.9	3.5
18	NSCLC	ND	Lung	2.7	1	Yes	Imaging not available	PD/NE	1.1	2.1
Stratum 1, 17 Gy × 1										
19	Melanoma	ND	Subcutaneous nodule	3	2	No	−18.20%	PD	1	2.1
25	NSCLC	ND	Lung	7.5	5	No	Unable to evaluate (RT changes)	PD	1.8	4.3
27	Melanoma	ND	Nodal	105.4	6 (+24)^e^	No	−43.60%	PR	28.1 ^f^	28.1^f^
32	NSCLC	ND	Sternal mass	204.2	2	No	1.40%	PD	1.4	1.4
41	NSCLC	ND	Scalp	3.9	2	Yes	−5.00%	PD	1.6	22.5^f^
42	Melanoma	30/15	Lung	3.3	6	No	−26.10%	PD	5.1	20.5^f^
Stratum 2, 8 Gy × 3										
1	Pancreas	ND	Liver	22.9	1	No	4.30%	PD	0.8	1.5
3	Adenoid cystic carcinoma (head and neck)	0/0	Lung	1.9	6	No	−10.50%	SD	7.4	34.4^f^
4	breast ER+/PR−/HER2−	ND	Bone	33.2	5	No	Unable to evaluate (bone lesion)	PD	3.1	9.1
5	Pancreas	ND	Liver	9.5	4	No	images not available	PD	2.7	3.1
6	Breast ER+/PR+/HER2−	ND	Liver	17	5	No	3.00%	PD	3	4.7
8	Pancreas	ND	Liver	47	2	No	17.60%	PD	1.2	6.3
Stratum 2, 17 Gy × 1										
9	RCC	0/0	Lung	3.4	6 (+14)^e^	No	−5.00%	SD	7	22
10	Breast ER+/PR+/HER2−	ND	Liver	4	2	No	−10.00%	PD	1.4	20.4
11	Pancreas	ND	Liver	19.9	1	No	Images not available	PD/NE	1	1.1
12	Colon	ND	Liver	64.1	4	No	Images not available	PD	2.1	12.1
13	Breast ER+/PR−/HER2−	ND	Liver	7.5	3	No	−12.50%	PD	1.9	6.9
16	RCC	ND	Lung	6.9	6 (+2)^e^	No	Unable to evaluate (RT changes)	CR	31.4^f^	31.4^f^

*ND* not determined

^a^See Methods for explanation; PD-L1 staining was only available on 4 patients. Most of the patients had their original diagnosis made outside Penn, and we were unable to have the original institution send samples to the third party that performed the staining

^b^Out of 6 intended cycles on protocol; number in parentheses indicates additional cycles of of pembrolizumab received off study (as protocol was amended so that paients who had completed 6 doses of pembrolizumab and were doing well could continue on the drug)

^c^Complete response (CR), partial response (PR), stable disease (SD), progressive disease PD), not evaluable (NE); 2 patients exhibited rapid PD after 1 month on treatment and for whom progression could not be documented from clinical exam or imaging. They were considered not evaluable (i.e., PD/NE). One patient was lost to follow-up at 6 weeks without any disease evaluation and was considered not evaluable (lost/NE)

^d^Maximum percent change in volume of irradiated lesion based on post-radiation scan compared with pre-radiation scan

^e^Number in parentheses after + indicates number of cycles of pembrolizumab given following 6 cycles given on protocol

^f^Indicates event free with continued follow-up

### Immune pharmacodynamics

**Fig. 3:**
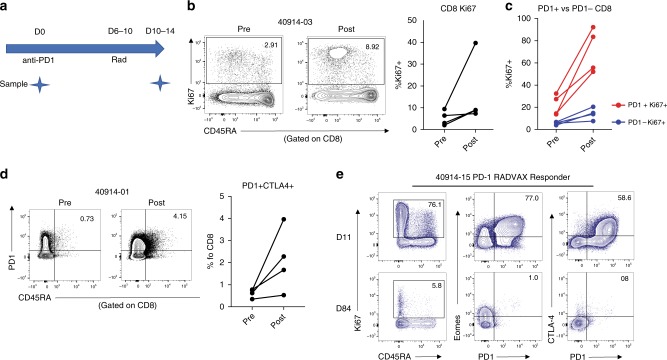
Pharmacodynamic immune response to anti-PD-1+ radiation. (**a**) Timeline of treatment and blood collections; D0 is pre-treatment and D10–14 is post RT. Panels **b**–**d** show flow cytometric data from 4 patients in stratum 2 treated with 8 Gy × 3 (patients 1, 3, 5, 6). (**b**) Ki67 in CD8 T cells pre- and post-RT from a representative stratum 2 patient and all 4 patients (**c**) Ki67 in PD-1+ versus PD-1− CD8 T cells pre- and post-RT. (**d**) Frequency of PD-1^+^CTLA4^+^CD8 T cells pre- and post-RT of a representative stratum 2 patient and all 4 patients (**e**) Phenotypic expression of CD8 T cells in the peripheral blood at days 11 and 84 of patient 15 (Fig. 2) who was treated with 8 Gy  × 3

We have recently shown that the pharmacodynamic immune response to anti-PD-1 therapy could be tracked in the peripheral blood using high-dimensional flow cytometry^21^. We applied this approach to a subset of patients treated using the 8 Gy × 3 radiation fractionation schedule who also had available samples at the pre-treatment and post-radiation time points. PBMCs were analysed using a pre-treatment sample (D0) and a post-radiation sample (D10–14) (Fig. 3a) to determine the pharmacodynamic immune effect of anti-PD-1 therapy (given D0) and radiation (given days 6–10). We tracked the immune response of four patients from stratum 2 who had breast cancer, pancreatic cancer and head and neck adenoid cystic carcinoma (patients 1, 3, 5, 6) and were all immunotherapy naive (Table 2). Treatment with pembrolizumab+RT resulted in an increase in PD-1^+^ CD8 T cells that were Ki67^+^, suggesting an on-target effect (Fig. 3b,c). Exhausted T cells, which are common in human tumours, express high levels of the transcription factor Eomes as well as multiple inhibitory receptors including CTLA4^21,22^. In these four patients, the frequency of PD-1^+^CTLA4^+^ CD8 T cells also increased with therapy, consistent to what we had shown with anti-PD-1 monotherapy^21^ (Fig. 3d).

We also tracked the immune response for patient 15 in stratum 1 who had a very good partial radiologic response to pembrolizumab and radiation after progression was documented by CT scan 6 months into nivolumab therapy (Fig. 2). We observed a large population of Eomes^hi^PD-1^+^CTLA4^+^-exhausted phenotype CD8 T cells (Tex) at an early post-treatment timepoint in this clinical responder (Fig. 3e). Altogether, this suggests that radiation may play a role in modulating the reinvigoration of exhausted CD8 T cells by anti-PD-1 therapy.

## Discussion

The combination of HFRT and pembrolizumab was well tolerated and feasible to administer using immune stimulatory dosing of either 8 Gy for three fractions or a single 17 Gy fraction, with responses observed in patients with prior progression on PD-1 blockade. Given the current context of several single-arm clinical trials of radiation and checkpoint blockade, our study is highly unique and novel in that it required patients with melanoma and lung cancer to have disease progression on PD-1 blockade prior to study entry. Given the well-known expected response rates from PD-1 blockade in these cancers, synergy with RT cannot be demonstrated in a single-arm trial of anti-PD-1 treatment-naive patients. We therefore chose to study the combination in patients with progression on PD-1 blockade, which is currently the most urgent unmet clinical need for improving patient outcomes. Our study is the first to prospectively enrol patients with disease progression on PD-1 blockade onto a study of checkpoint blockade and RT to assess the effect of RT on the response to immune therapy.

The selected treatment plan was well tolerated. All the toxicities on our trial attributed to pembrolizumab and/or radiation were grade 1 or 2. Out of 24 patients on our trial, 17 (71%) had at least one drug-related AE, which is in line with results from large clinical trials^23,24^. While the study was not powered for a formal comparison, both schedules of radiation with pembrolizumab on our trial appear to be safe (Table 2). Pembrolizumab can be associated with various adverse immune effects such as pneumonitis, colitis and endocrinopathies^25^. We saw no evidence that the addition of radiation to pembrolizumab increased the rate of immune-related toxicity since only one patient developed these types of toxicities. Of note, we enrolled only patients with the Eastern Cooperative Oncology Group (ECOG) performance status of ≤1, which may have contributed to our low incidence of grade 3 or higher toxicity. Our rationale for this was that we wanted to have patients who would have long enough survival for us to assess an abscopal effect of radiation, which was a secondary endpoint.

A group from the University of Chicago recently published the results of their study with pembrolizumab and SBRT in patients with metastatic cancer and reported that 6 out of 62 subjects had treatment-related grade 3 or higher toxicities (9.7%)^26^. We did not observe any such grade 3 or higher treatment-related toxicities, but there were key differences between their trial and ours. The University of Chicago group also enrolled patients with an ECOG performance status ≤1, but they used doses ranging from 30 Gy in 3 fractions for osseous disease to 50 Gy in 5 fractions for central lung and mediastinal disease and 45 Gy in three fractions for other sites, whereas we used either 24 Gy in three fractions or a single dose of 17 Gy. They also treated at least two sites of metastases, whereas in our trial only a single site was irradiated. They noted that when toxicity was observed, it appeared to be in the region that was irradiated, making it difficult to distinguish between toxicity of combination therapy versus radiation or pembrolizumab alone.

The inclusion of prospectively enrolled patients who had previously progressed on anti-PD-1/PD-L1 therapy was novel and an important distinguishing element in our study design. We reasoned that if these patients showed evidence of tumour shrinkage in spite of prior progression, it is likely that the radiation played a role. Two patients in this stratum experienced a PR that was durable, suggesting that in a subset of patients, radiation can increase the immune response to pembrolizumab. These findings are consistent with prior retrospective work which noted an abscopal effect for patients receiving ICB who also received RT^27^. Furthermore, while atypical or delayed responses are observed in patients treated with pembrolizumab, the rate of delayed immune responses in a study of 327 patients treated with pembrolizumab was only 7%, most of which were detected at the second response assessment^28^. Conversely, our study patients who achieved partial responses after progression on PD-1 blockade had been treated for 8 and 10 months, respectively, prior to study entry, strengthening the synergistic contribution of RT in effecting an anti-tumour immune response, as opposed to a delayed or atypical immune response which is rare with PD-1 blockade.

In focussing on the two patients with prior progression on PD-1 blockade who had PRs, we found no consistent clinical characteristics that distinguished them from the non-responders. Both patients had nodal masses that were irradiated; however, in patient 27 the mass was quite large (105 cm^3^), whereas in patient 15 it was small (16 cm^3^). The doses of radiation used for these patients were also different, 8 Gy × 3 for patient 15 versus 17 Gy × 1 for patient 27. The rationale of using HFRT was based on pre-clinical and clinical reports that had used similar fractionation schemes^13,29^. A recent study suggests that doses greater than 12–18 Gy might actually attenuate immunogenicity by degrading DNA that accumulates in the cytosol after irradiation^30^. However, we very clearly saw an abscopal response in patient 27 who received 17 Gy × 1.

The previous discussion centres around the use of HFRT; however, it is noteworthy that in the recently published phase 3 randomised PACIFIC trial^31^, the group that received consolidation therapy with the anti-PD-L1 antibody durvalumab following standard chemoradiation for locally advanced lung cancer had a much better PFS than the placebo group. The radiation therapy regimen was 54–66 Gy in conventional fractionation. If this improvement in PFS was the result of some interaction between radiation and durvalumab, then it begs the question of whether HFRT is required to elicit an immune response with ICB and whether conventional radiotherapy may do this better.

In stratum 2 we treated patients with a variety of histologies who had never previously received anti-PD-1 or anti-PD-L1 therapy. None of the patients with pancreatic cancer (*n* = 4) or breast cancer (*n* = 4) showed any clinical or radiologic benefit as they all had progressive disease, which occurred fairly rapidly in several patients. Pancreatic cancer has not been a histology that has shown any response to pembrolizumab, and adding radiation did not seem to help in this trial. The only subtype of breast cancer that has shown response to pembrolizumab is triple-negative breast cancer (overall response rate 18.5% in KEYNOTE-012)^32^; however, among our four patients with breast cancer, only one had this subtype. Of note, all the patients with cancer of the breast or pancreas had received previous chemotherapy, sometimes multiple courses (Supplementary Table 1)

Also in stratum 2, one patient with renal cell carcinoma experienced a CR and another experienced SD. At the time of trial design, anti-PD-1 therapy was not FDA approved for the management of renal cell carcinoma, but we now know that this therapy has significant activity^33^. There was also a patient with adenoid cystic carcinoma who had SD as best response and so may have derived some clinical benefit. Preliminary data indicate that salivary gland cancers have an overall low response rate to PD-1 inhibition, but responses have been reported. It is therefore hard to know whether radiation added value to pembrolizumab in these cases.

We observed an expansion of PD-1^+^CTLA4^+^CD8 T cells after RT, consistent with recently published literature that this specific subset of cells may have an exhaustion phenotype and be responsible for anti-tumour immunity^21,22^. Of note, a significant T_EX_ population could be identified in a responder post-RT treatment, suggesting that radiation may play a role in augmenting the effect of anti-PD-1 therapy by modulating the reinvigoration of T_EX_. As our trial remains open and continues to enrol patients in the Expansion Phase, this study provides the opportunity for future studies to interrogate how radiation modulates reinvigoration of the immune response with anti-PD-1 therapies and the underlying mechanisms of resistance. Ultimately, a randomised trial is required to demonstrate synergistic benefit of RT with checkpoint blockade compared to checkpoint blockade alone; we have planned a randomised trial with activation expected in 2018.

## Electronic supplementary material

Supplementary Data
