# Supplementary material for: A phase I trial of pembrolizumab with hypofractionated radiotherapy in patients with metastatic solid tumours
Source: Br J Cancer. 2018 Oct 15;119(10):1200–7. doi: 10.1038/s41416-018-0281-9 (PMC6251028; doi:10.1038/s41416-018-0281-9)
Supplement: Supplementary file 1 — Supplementary Data [file 41416_2018_281_MOESM1_ESM.pdf]

Supplementary Figure 1

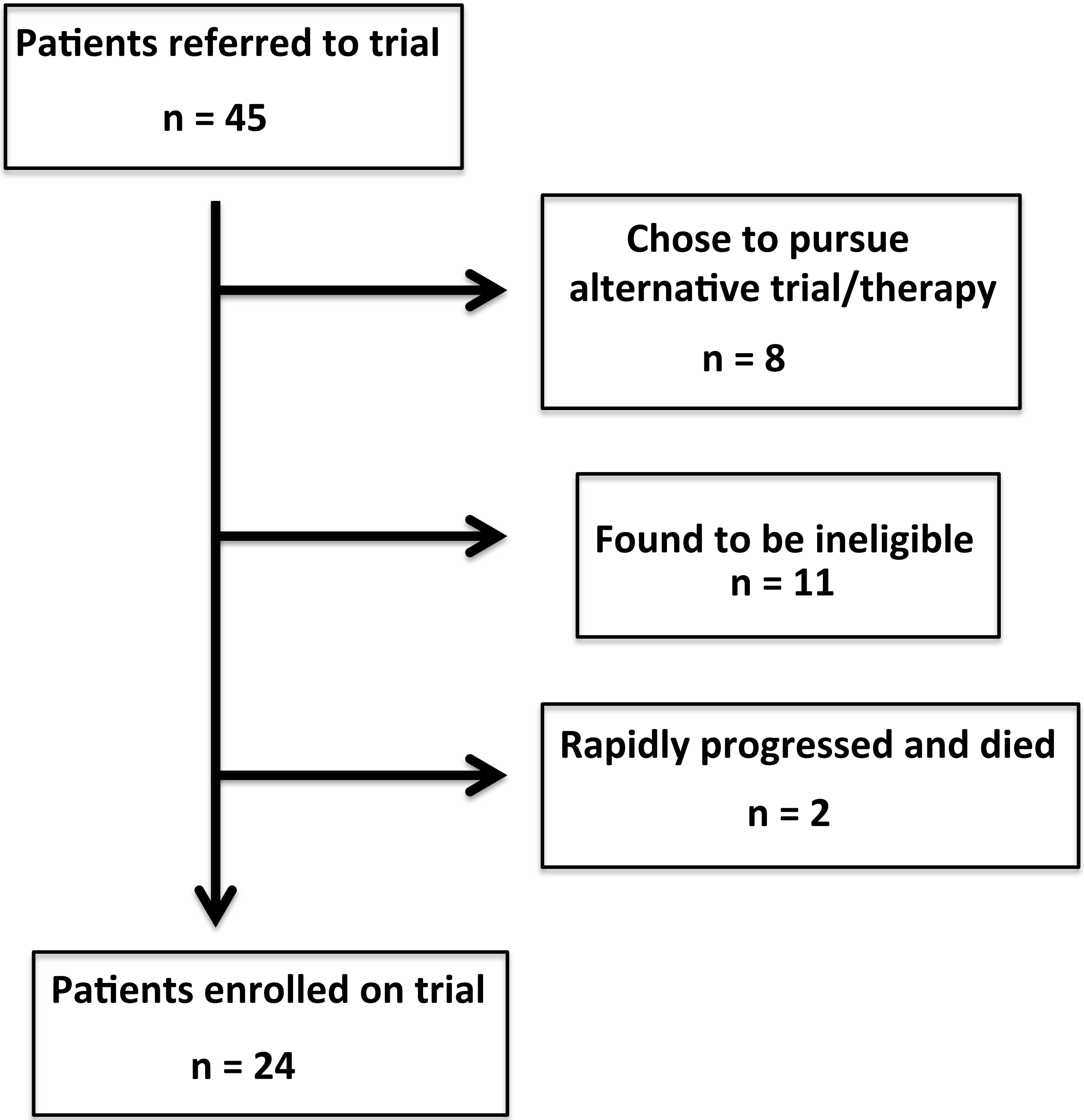

Suppl. Figure 2

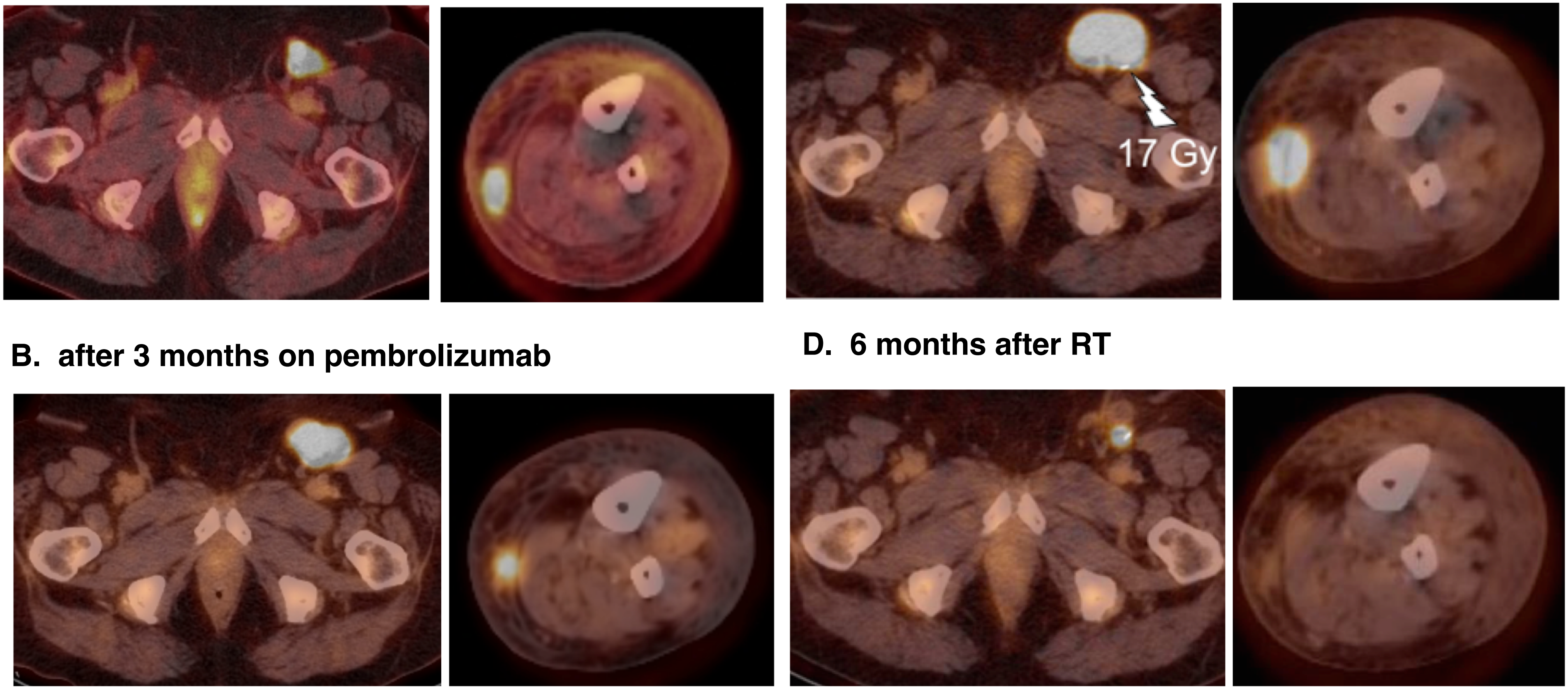

Patient 27 was diagnosed with melanoma of left foot. A year later he developed left inguinal adenopathy; biopsy showed melanoma. He recurred after 4 cycles of ipilimumab, both in the left inguinal region and in a subcutaneous nodule in the left calf. He had an FDG PET/CT scan (panel A) and was switched to pembrolizumab. PET/CT scan 3 months later showed a mixed response: decrease in size of calf lesion but increase in size of inguinal mass (panel B). By 9 months he had clear progression of disease in both lesions (panel C). Therefore, he was enrolled on the RadVax trial. He received pembrolizumab followed by 17 Gy x 1 to the inguinal mass. Panels C shows baseline PET/CT scan at start of trial, and panel D shows scan six months after RT.

Supplementary Table 1 Previous systemic therapies

**Stratum 1**				
**Patient #**	**1° site**	**Previous systemic therapies**	**# cycles of anti-PD-1 therapy prior to enrolling on RadVax trial**	**Criteria for considering progression**
**2**	NSCLC	carboplatin/gemcitabine, carboplatin/paclitaxel, carboplatin/Nab-paclitaxel, pembrolizumab	22 pembrolizumab	new R adrenal lesion
**7**	melanoma	ipilimumab, pembrolizumab	4 pembrolizumab	new bilateral sclav, R axilla, L hilar, R paraspinal, L abdominal wall lesions
**14**	melanoma	ipilimumab/nivolumab, pembrolizumab	8 nivolumab/3 pembrolizumab	new L axillary nodes
**15**	NSCLC	carboplatin/paclitaxel, gemcitabine/vinorelbine, nivolumab	17 nivolumab	new portocaval adenopathy
**17**	NSCLC	carboplatin/paclitaxel, nivolumab	6 nivolumab	new R hepatic, R kidney, mesenteric lesions
**18**	NSCLC	vinorelbine/cisplatin, paclitaxel/carboplatin, gemcitabine/docetaxel, nivolumab	8 nivolumab	new R occipital lobe metastasis
**19**	melanoma	Ipilimumab, pembrolizumab	11 pembrolizumab	new splenic and liver lesions
**25**	NSCLC	pemetrexed,carboplatin/Nab-paclitaxel, etirinotecan pegol, nivolumab	5 pembrolizumab	44% increase lesions by RECIST
**27**	melanoma	ipilimumab, pembrolizumab	12 pembrolizumab	39 % increase lesions by RECIST
**32**	NSCLC	cisplatin/gemcitabine, nivolumab	5 pembrolizumab	new pleural masses
**41**	NSCLC	paclitaxel/carboplatin, pemetrexed/carboplatin, gefitinib, nivolumab	5 pembrolizumab	new pleural masses
**42**	melanoma	ipilimumab, pembrolizumab	13 pembrolizumab	new R lung nodule
**Stratum 2**				
**Patient #**	**1° site**	**Previous systemic therapies**		
**1**	pancreas	gemcitabine/Nab-paclitaxel/capecitabine, sorafenib	NA	NA
**3**	Head and neck	None	NA	NA

	(adenoid cystic Ca)			
**4**	breast	paclitaxel, anastrozole, exemestane, tamoxifen, docetaxel, bevacizumab, fulvestrant, capecitabine, cyclophosphamide, gemcitabine, eribulin,	NA	NA
**5**	pancreas	gemcitabine/Nab-paclitaxel	NA	NA
**6**	breast	capecitabine, Nab-paclitaxel	NA	NA
**8**	pancreas	gemcitabine /Nab-paclitaxel, FOLFIRINOX, oxaliplatin	NA	NA
**9**	RCC	sorafenib, sunitinib, panzopanib, everolimus, axitinib,	NA	NA
**10**	breast	tamoxifen, paclitaxel, anastrozole, fulvestrant, exemestane/everolimus, capecitabine	NA	NA
**11**	pancreas	gemcitabine/Nab-paclitaxel, FOLFIRINOX, erlotinib	NA	NA
**12**	colon	FOLFIRINOX /bevacizumab, 5FU/leukovorin	NA	NA
**13**	breast	docetaxel, doxorubicin/cyclophosphamide, capecitabine, letrozole, exemestane, gemcitabine, eribulin, ixabepilone	NA	NA
**16**	RCC	none	NA	NA

NA – not applicable

Supplementary Table 2 All adverse events by stratum and fractionation

Type of Toxicity	Stratum 1				Stratum 2			
	8 Gy x 3 n=6		17 Gy x 1 n=6		8 Gy x 3 n=6		17 Gy x 1 n=6	
	1-2	3	1-2	3	1-2	3	1-2	3
**Cardiac:**								
Chest Pain	2		1					
Palpitations							1	
**Endocrine:**					1			
Hypothyroidism							1 (P)	
**Gastrointestinal:**								
Abdominal pain/cramping	2 (2R)				3		1	
Bloating	1		1		1		1	
Bowel Obstruction				1				
Constipation	1 (P)		4 (3P)		1			
Diarrhea			3 (3P)				1 (P)	
Dry Mouth			1					
Dyspepsia	1 (P)				1 (P)			
Fecal Incontinence	1							
Flatulence			1					
Nausea	3 (2P, 1R)*		1		2 (2P)		1	
Vomiting	2 (1P, 1R)		1		1			
**General Disorders**:								
Chills							1	
Fatigue	2 (1P)		3 (3P)		2 (1P)		4 (1P)	
Fever			1 (1P)		2 (1P)	1 (P)	2 (1P)	
Generalized aches					1 (1P)			

Peripheral edema	2		2		1		1	
Weakness	1	1	1 (P)		1		1 (P)	1
**Infections**								
Cellulitis			1					
Bacteremia						1 (likely GI source)		
Sinusitis	1							
Upper Respiratory Infection					1		1	
Urinary Tract Infection	1						1	
**Injury:**								
Bruising					1			
**Metabolic & Nutritional**:								
Anorexia	2		1		2			
Dehydration		1	1		1			
**Musculoskeletal**:								
Arthralgia	1 (P)							
Back pain	1				1		1	
Bone pain	1							
Chest wall pain			1					
Muscle cramping							1	
Myalgia							1	
Scalp pain	1 (R)							
**Nervous System:**								
Dizziness	1 (P)				1			
Dysgeusia	1		1					
Headache	1 (P)		1 (P)		1 (P)		1 (P)	
Lightheadedness	1							
Migraine							1	
Neuralgia	1							
Paresthesias	2		1		1		2	
Sciatica					1			

**Psychiatric:**								
Anxiety	1		1					
Confusion		1	2					
Depression	2		1				1	
Insomnia	2 (1P)		2					
**Respiratory:**								
Cough			3		1		2	
Dyspnea			2				1	
Hoarseness	2							
Pleural Effusion			1					
Pneumonitis							1 (P and R)	
Wheezing	1				1			
**Skin:**								
Pruritus	1 (P)				1		1	
Rash							2 (1P)	
**Vascular:**								
Edema	2		3		1		1	
Flushing			1 (P)					
Hot flashes					1			
Hypotension	1			1				
Thromboembolic event					1 (deep venous thrombosis)			

* Number within parentheses indicate attribution. For example, 3 (2P, 1R) indicates that out of the three events, two were attributed to pembrolizumab and one was attributed to radiation. No parentheses indicate events were not attributed to pembrolizumab or radiation.
